# Does Long-term Soft Contact Lens Wear Affect Corneal and Anterior Chamber Parameters?

**DOI:** 10.4274/tjo.53486

**Published:** 2018-09-04

**Authors:** Cemal Çavdarlı, Pınar Topçu-Yılmaz

**Affiliations:** 1 University of Health Sciences, Ankara Numune Training and Research Hospital, Ophthalmology Clinic, Ankara, Turkey

**Keywords:** Soft, contact lens, cornea, anterior chamber, topography

## Abstract

**Objectives:**

To assess the long-term effects of soft contact lenses (SCL) on the cornea and anterior chamber by topography.

**Materials and Methods:**

Thirty-nine eyes of 22 healthy patients were included in this prospective study. Changes in corneal and anterior chamber parameters before and after 12 months of daily SCL use (Air Optix Aqua, Air Optix Aqua for Astigmatism, Acuvue Oasys and Acuvue Oasys for Astigmatism) were evaluated with Pentacam (Oculus, Germany).

**Results:**

Best corrected visual acuity with toric SCL was significantly better compared to spectacles in the toric SCL group (0.98±0.34 vs 0.94±0.72, p=0.004). None of the corneal (horizontal and vertical keratometry, corneal volume, anterior and posterior corneal astigmatism, corneal pachymetry of apex and thinnest location) and anterior chamber (anterior chamber depth, volume and angle) parameters showed a statistically significant change after long-term daily wear of SCLs.

**Conclusion:**

The results of this study revealed that long-term wear of current high oxygen permeable and relatively low modulus silicone hydrogel SCLs does not impact cornea and anterior chamber morphology or volumetric parameters. Furthermore, toric silicone hydrogel SCLs can provide better visual performance than spectacles.

## Introduction

Soft contact lenses (SCL) are currently one of the most popular modes of refractive error correction. With the advances in contact lens technology, they have become more comfortable and safer; however, they still carry the risks associated with modification of corneal morphology. The primary goal in SCL design is to create a contact lens which provides biocompatibility with both the cornea and the surrounding ocular environment (e.g. tear film, conjunctiva, eyelids). The mechanical properties of the SCL (edge design, diameter, modulus) should not increase corneal and conjunctival epithelial irritation and should allow maximum tear circulation.^1^

Despite the developments in contact lens technology, the alterations in corneal metabolism and the mechanical forces associated with contact lens wear can affect the anterior shape of the cornea and result in central corneal steepening or flattening, loss of radial symmetry, and changes in astigmatism or optical higher order aberrations.^2,3,4,5,6^ Significant improvements in silicone hydrogel lenses have made them the first choice for new contact lens wearers.^7^ Silicone hydrogel lenses have eliminated corneal hypoxia.^8^ However, despite their high oxygen transmission levels, SCL-related central and peripheral corneal swelling which results in increased central corneal thickness and significant thinning of the central corneal epithelium has been reported.^9,10^

Both the anterior corneal surface and corneal thickness are critical variables to consider before any clinical intervention, particularly refractive surgery. Furthermore, changes in the posterior corneal surface are useful in monitoring corneal pathologies and in the early detection of corneal ectatic disorders.^11^ Thus, monitoring changes in the anterior and posterior cornea is important in contact lens wearers who are candidates for keratorefractive surgery.

The purpose of this study was to assess changes in corneal and anterior chamber parameters after 12 months of SCL wear.

## Materials and Methods

Thirty-nine eyes of 22 individuals who attended the contact lens clinic of Ankara Numune Training and Research Hospital between January 2014 and January 2016 were included in this prospective study. Patients with known history of ocular and/or systemic diseases, previous ocular surgery, prior contact lens use, ocular or systemic medication use, and more than 3.5 diopters of cylindrical and/or 6 diopters of spherical refractive error were not included. All subjects underwent complete ophthalmologic evaluation to ensure the presence of Snellen best corrected visual acuity of 20/25 or better, normal anterior and posterior segment biomicroscopy, normal tear film functions (Schirmer test >15 mm/5 minute and tear break-up time ≥10 second) and  normal intraocular pressure. Informed consent was obtained from all subjects and the study was carried out with approval from the ethics committee of Ankara Numune Training and Research Hospital.

Refractive and topometric maps obtained with Pentacam (Oculus, Germany) were used to evaluate anterior and posterior corneal axial curvature, corneal volume, central corneal thickness, corneal thickness at the thinnest point, anterior chamber depth, anterior chamber angle, and anterior chamber volume before contact lens fitting. All measurements were performed by a single author (C.C.) between 10:00 and 12:00 a.m. to avoid diurnal variations. Only reliable scans that marked “quality specification” of Pentacam were included in analysis.

After the completion of Pentacam measurements, each patient was fitted with a suitable Lotrafilcon B (Air Optix Aqua, Air Optix Aqua for Astigmatism, CIBA Vision, Duluth, USA) or Senofilcon A (Acuvue Oasys, Acuvue Oasys for Astigmatism, Johnson&Johnson, Jacksonville, USA) SCL according to their manifest refraction. Subjects were instructed to wear these contact lenses in daily wear mode before returning for the final visit at 12 months. The cornea and anterior chamber were re-evaluated with Pentacam (Oculus, Germany) at the final visit, at least 1 hour after contact lens removal to avoid lens-related mechanical changes. Subjects who wore their contact lenses regularly (>4 days/week) in daily wear mode were included in the final analysis.

### Statistical Analysis

Statistical analyses were performed using SPSS version 13.0 (Chicago, IL, USA) software. Student’s paired t-test was used to compare changes in visual acuity, keratometry, corneal volume, central corneal thickness, corneal thickness at thinnest point, anterior chamber depth, anterior chamber angle, and anterior chamber volume over time. P value less than 0.05 was considered to be statistically significant.

## Results

Thirty-nine eyes of 22 patients were initially included in the study, but 2 of the subjects were excluded due to irregular SCL wear. Thus, the study was completed with a total of 37 eyes of 20 patients (12 female, 8 male). Mean age was 20.52±3.18 years (range 15-28 years). Nineteen eyes were fitted with spherical soft lenses and 18 eyes were fitted with toric soft lenses.

Best corrected distance visual acuity (BCVA) for patients fitted with spherical lenses was 20/20 at baseline and final examination. There was a statistically significant increase in the distance BCVA of toric lens wearers (0.94±0.72 vs 0.98±0.34, p=0.004) (Table 1), and the significance in the total group seems to originate from the toric group.

The corneal (keratometry, corneal volume, anterior and posterior astigmatism, corneal apex, and thinnest-point corneal pachymetry) and anterior chamber parameters (anterior chamber depth, anterior chamber volume, and chamber angle) as evaluated by Pentacam are summarized in Tables 2 and 3, respectively. None of these parameters showed a statistically significant change after long-term contact lens wear.

## Discussion

Contact lenses are a popular mode of refractive error correction due to advantages such as a larger field of vision, better optical quality, and cosmetic appeal. However, they still have the disadvantage of interfering with normal corneal physiology and curvature.^11^ The purpose of our study was to investigate the long-term effects of SCL wear on corneal and anterior chamber parameters measured by Pentacam. To our knowledge, this is the first prospective study to investigate long-term SCL-associated changes in the cornea (keratometry, corneal volume, central corneal thickness, corneal thickness at thinnest point) and the anterior chamber (anterior chamber depth, angle, and volume) with Pentacam. This device has been found to be highly reliable and showed excellent repeatability in previous studies.^12,13^

Snellen best corrected visual acuity of 20/20 was our targeted value for SCL wearers when prescribing contact lenses. Thus, some of the patients might have better visual acuity after SCL fitting. While we were unable to find a significant difference in the visual acuity of spherical lens wearers, a more detailed visual acuity study could also reveal significance in the spherical group. Visual performance in toric lens wearers was better with SCLs compared to spectacles. This result is in line with previous studies that have found improved visual quality and acuity with toric SCLs in low and moderate astigmatic eyes.^14,15^ We believe that with a good SCL-cornea adaptation process, the mechanical properties of contact lenses and the toric designs result in fewer aberrations with toric lenses compared to glasses.

Several studies have investigated the changes in corneal shape and thickness associated with contact lens wear. However, the follow-up for most of these studies was very short. Liu and Pflugfelder^16^ evaluated corneal thickness and topography in long-term contact lens wearers and healthy controls. They found that corneal thickness was significantly reduced by 30-50 µm in contact lens wearers. Furthermore, corneal curvature and surface irregularity were increased in eyes wearing contact lenses. Yeniad et al.^17^ evaluated changes in corneal curvature and thickness after 1, 6, and 18 months of rigid gas-permeable and soft lens wear. SCL wearers in their study showed corneal thickening at 1 month, followed by corneal thinning at 6 and 18 months. An increase in radius of corneal curvature and central corneal thinning associated with continuous wear of first-generation high-Dk SCLs were previously reported in a small sample of patients.^18^

Contrary to these studies, Alba-Bueno et al.^2^ compared corneal topographical changes with 3 months daily wear of silicone hydrogel (SiH) lenses and found that corneal topographic indices were stable with daily wear of SiH lenses. The magnitude of corneal morphological changes associated with 8 hours wear of daily disposable lenses was also small.^19^ Radaie-Moghadam et al.^20^ investigated corneal hysteresis, corneal resistance factor, central corneal thickness, and keratometry on toric SCL wearers with negative history of SCL wear and concluded that corneal hysteresis and corneal resistance factor decreased at 1 month and returned to baseline after 3 months, while central corneal thickness and corneal curvature values did not change significantly.

In the present study; Acuvue Oasys, Acuvue Oasys for Astigmatism, Air Optix Aqua, and Air Optix Aqua for Astigmatism were selected to evaluate the presumptive corneal alterations. These SCLs constitute the majority of SCL prescriptions in our clinic. The mean CCT was 553.64±23.22 at baseline and none of the corneal parameters showed significant difference after 12 months of SiH contact lens wear. Conflicting results in different studies are most likely due to differences in contact lens material, oxygen transmissibility, water content, and modulus. The results of our study show that low modulus, high oxygen transmissible lenses can prevent the corneal alterations associated with SCL use. Early corneal biomechanical and curvature changes which develop in the first few months of SCL wear regress in long-term follow-up after the cornea-contact lens adaptation process.

This study also investigated changes in anterior chamber volume, depth, and angle with SCL wear. As expected, no significant changes were observed in anterior chamber parameters.

### Study Limitations

This study has several limitations. First, our sample size was relatively small and did not allow for segregation of patients into subgroups depending on the modulus of lens materials.

Another limitation is the lack of aberrometry measurements, which would be helpful to explain the improvement in visual acuity in the toric SCL group.

## Conclusion

In conclusion, the results of this study revealed that previously reported short-term SCL-associated corneal changes probably diminish in long-term follow-up. Long-term wear of current highly oxygen permeable and relatively low modulus SiH SCLs do not change corneal or anterior chamber morphology and volumetric parameters. Furthermore, toric SiH SCLs may provide better visual performance compared to spectacles. Further studies with a larger sample size and longitudinal follow-up are necessary to confirm these findings.

## Figures and Tables

**Table 1:**
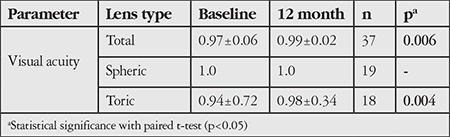
Snellen best corrected visual acuity at baseline and after 12 months of contact lenses wear

**Table 2:**
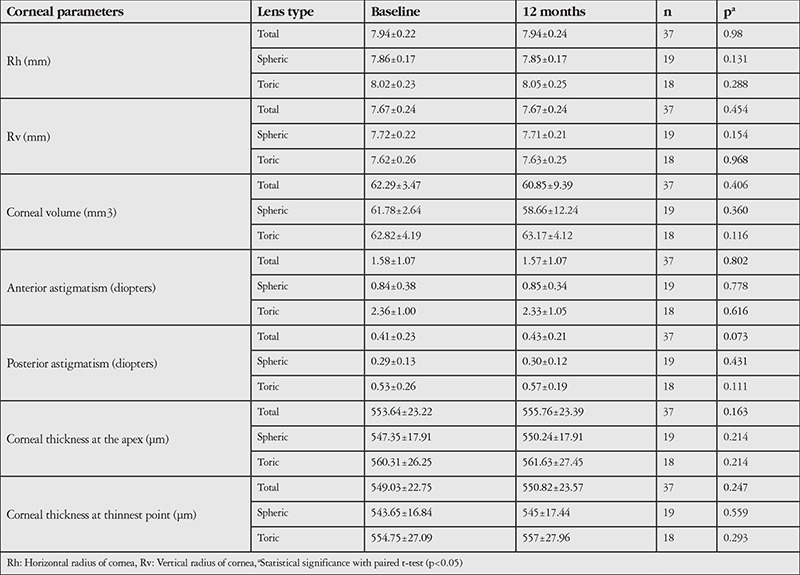
Evaluation of corneal parameters at baseline and 12-month follow-up

**Table 3:**
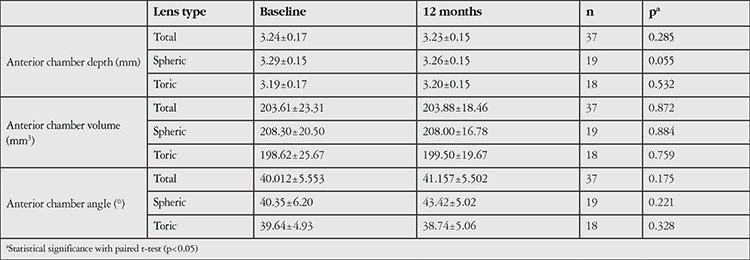
Evaluation of anterior chamber parameters at baseline and 12-month follow-up
